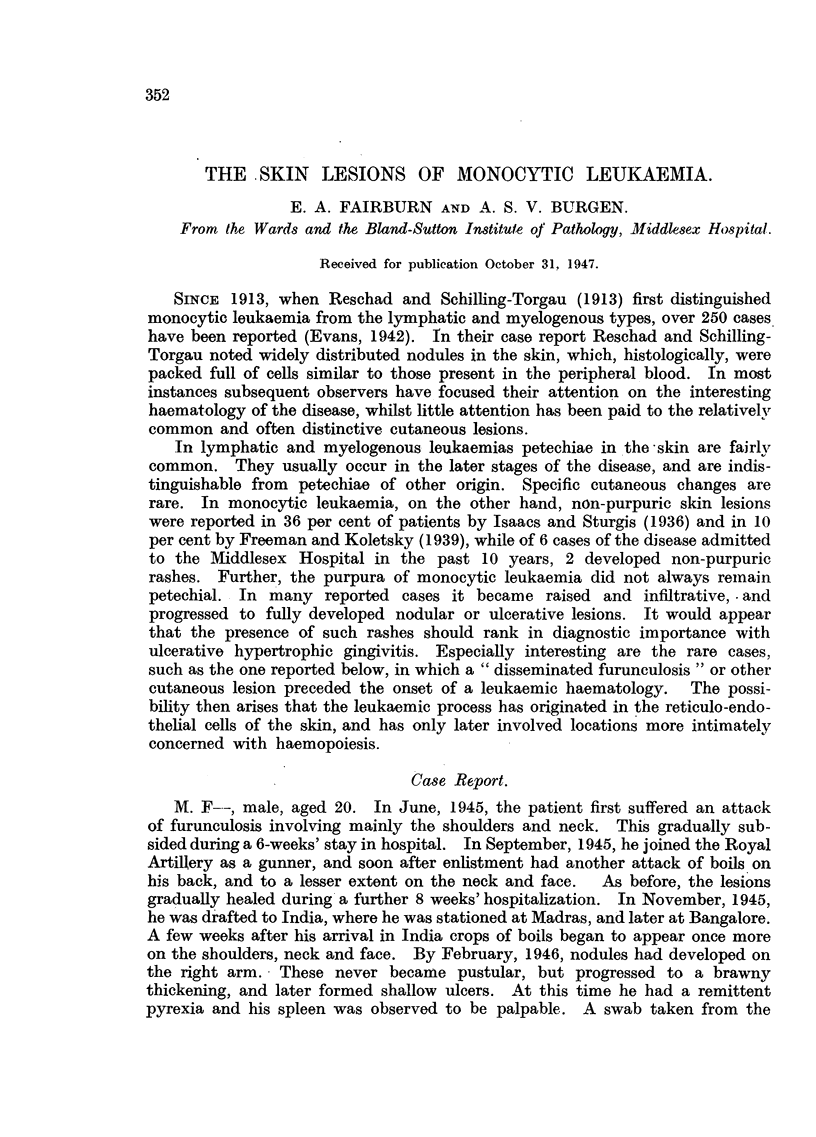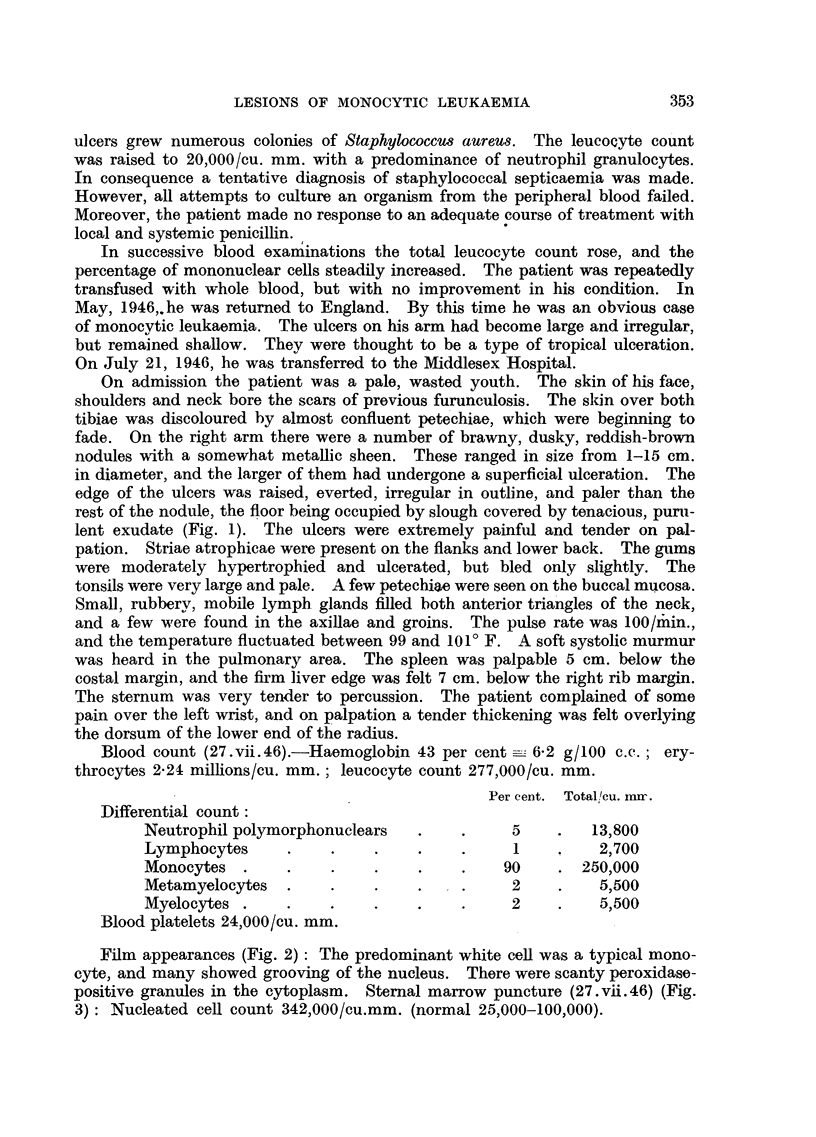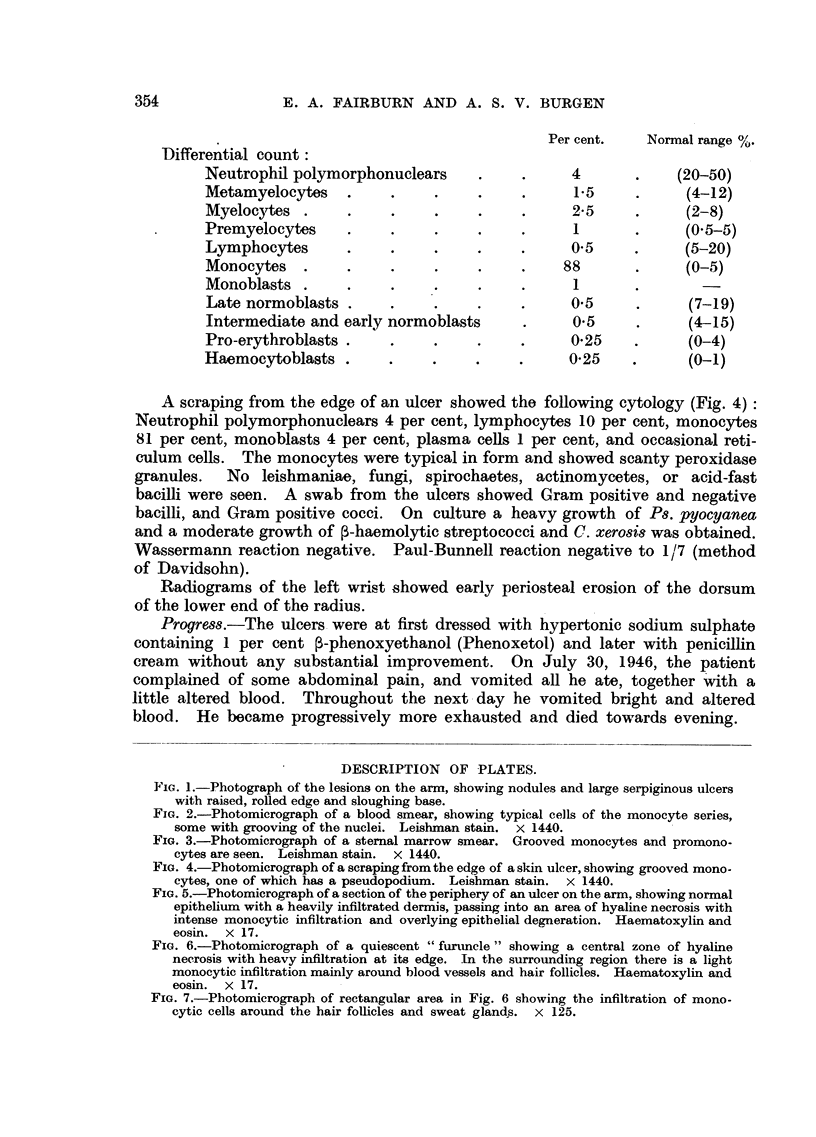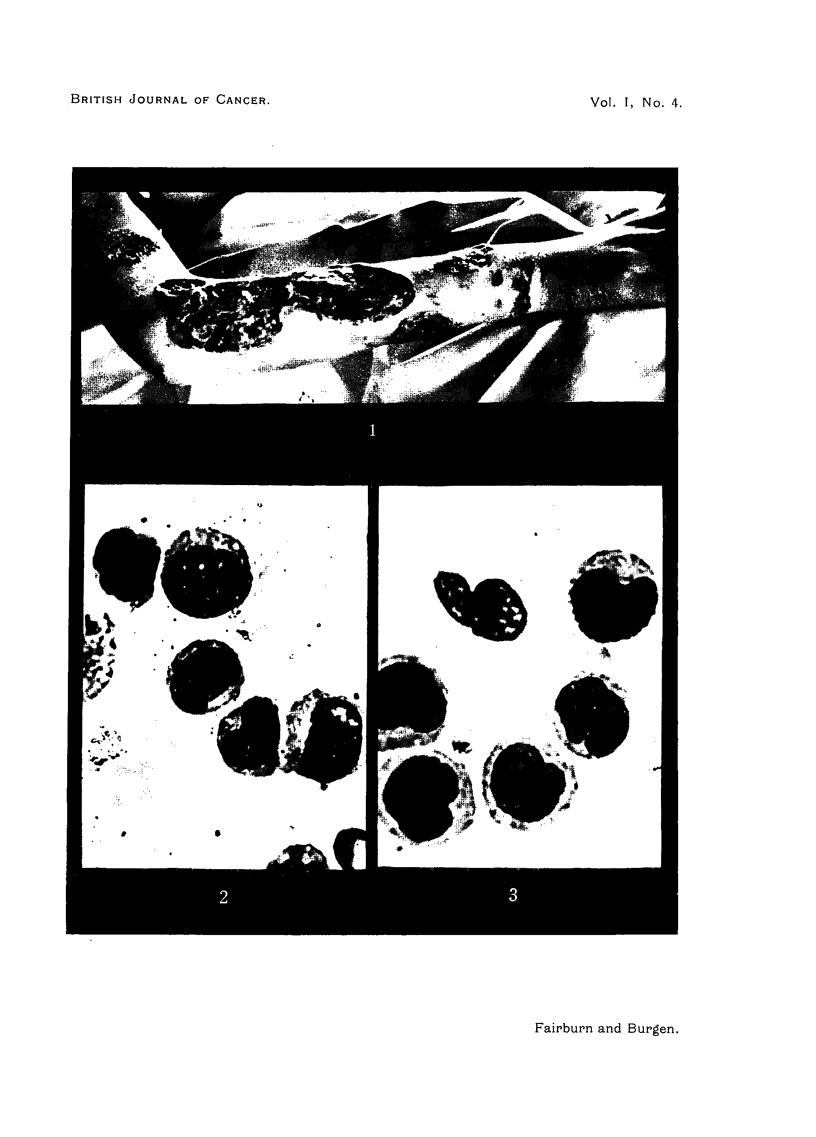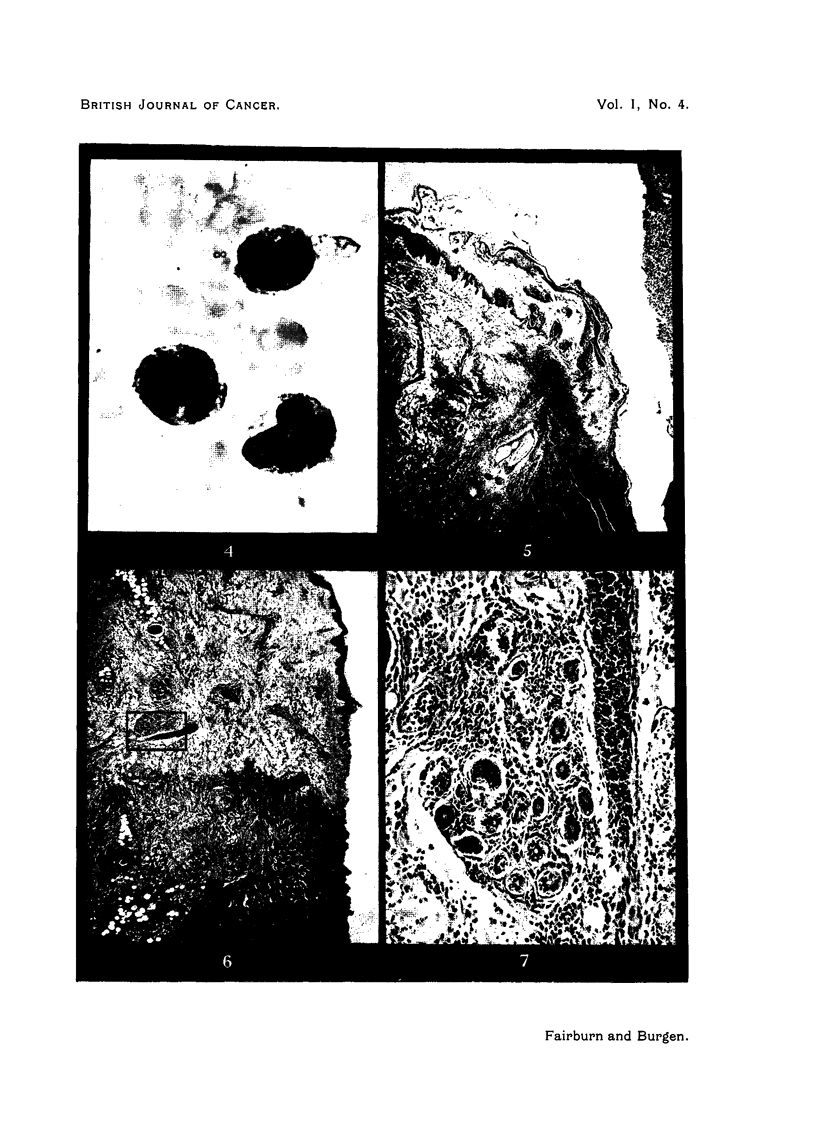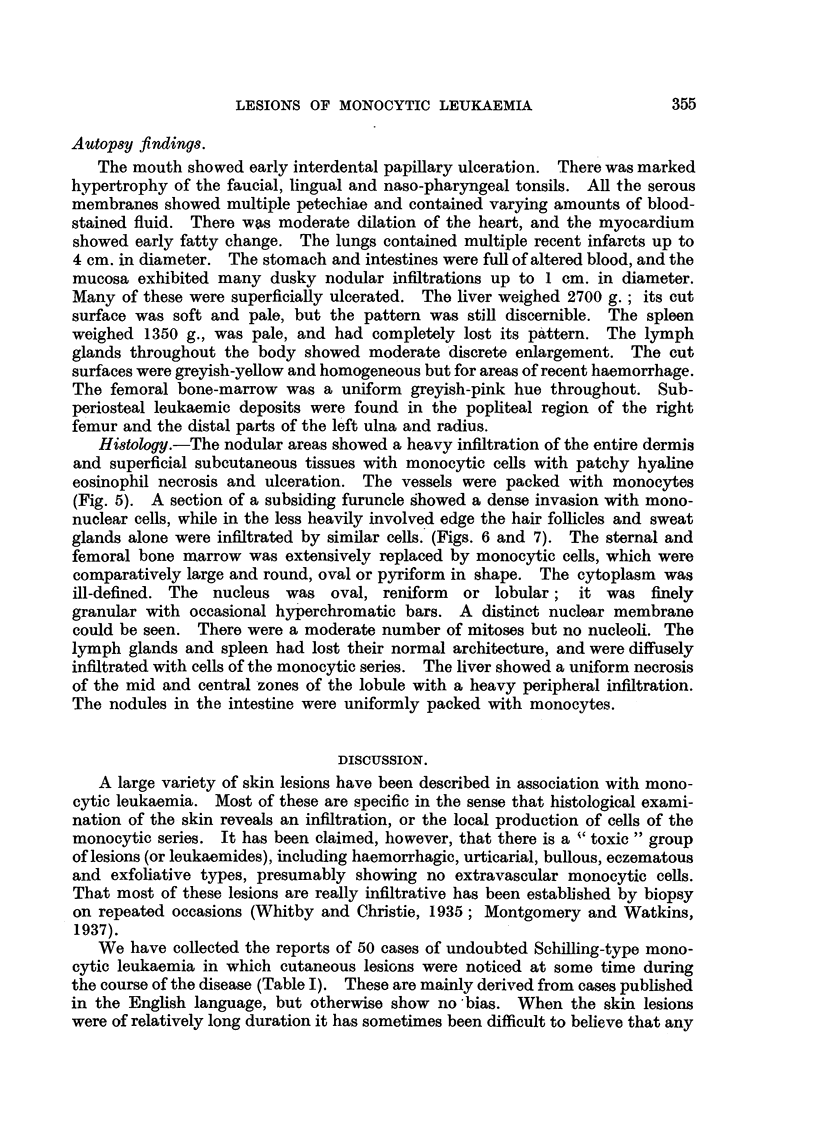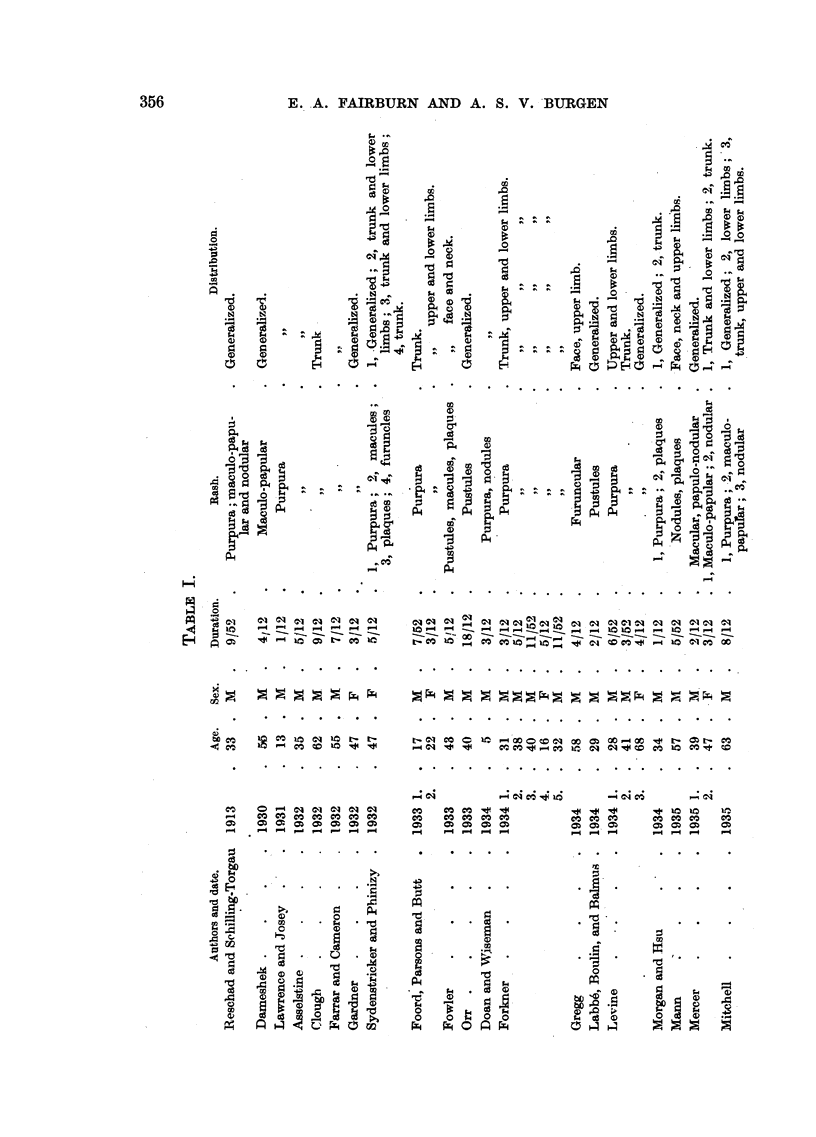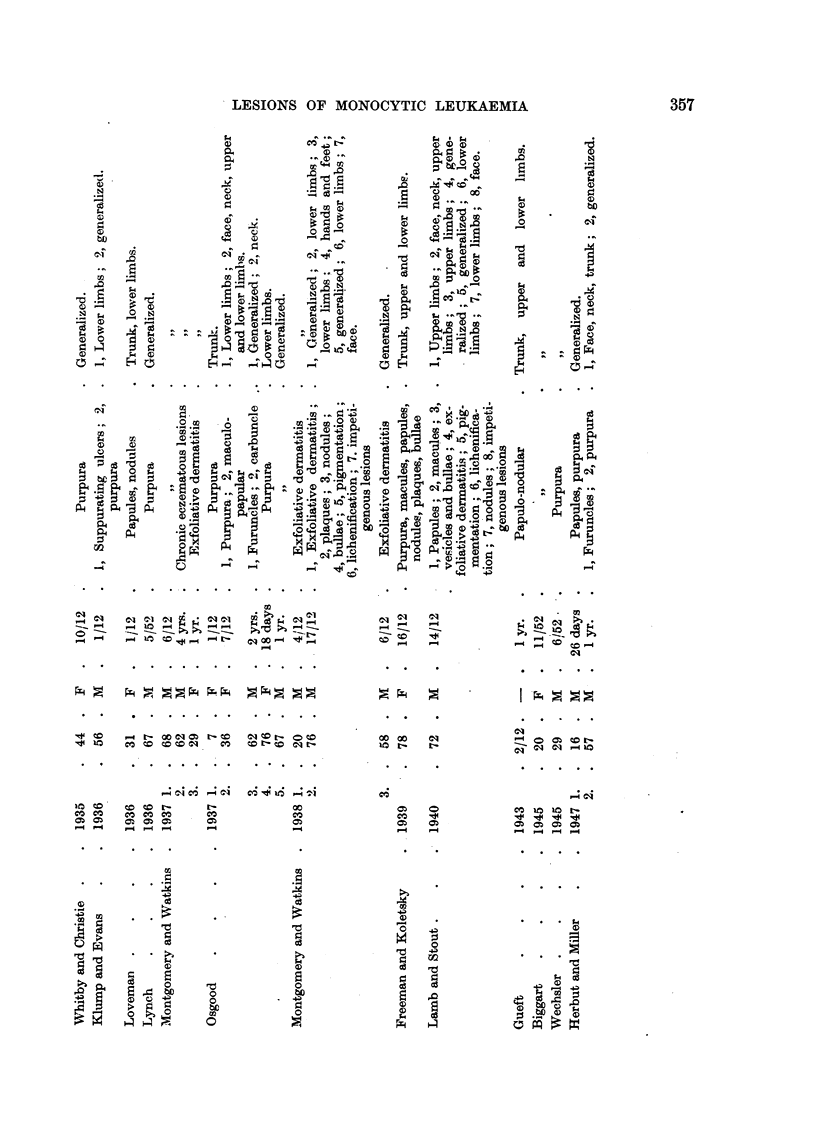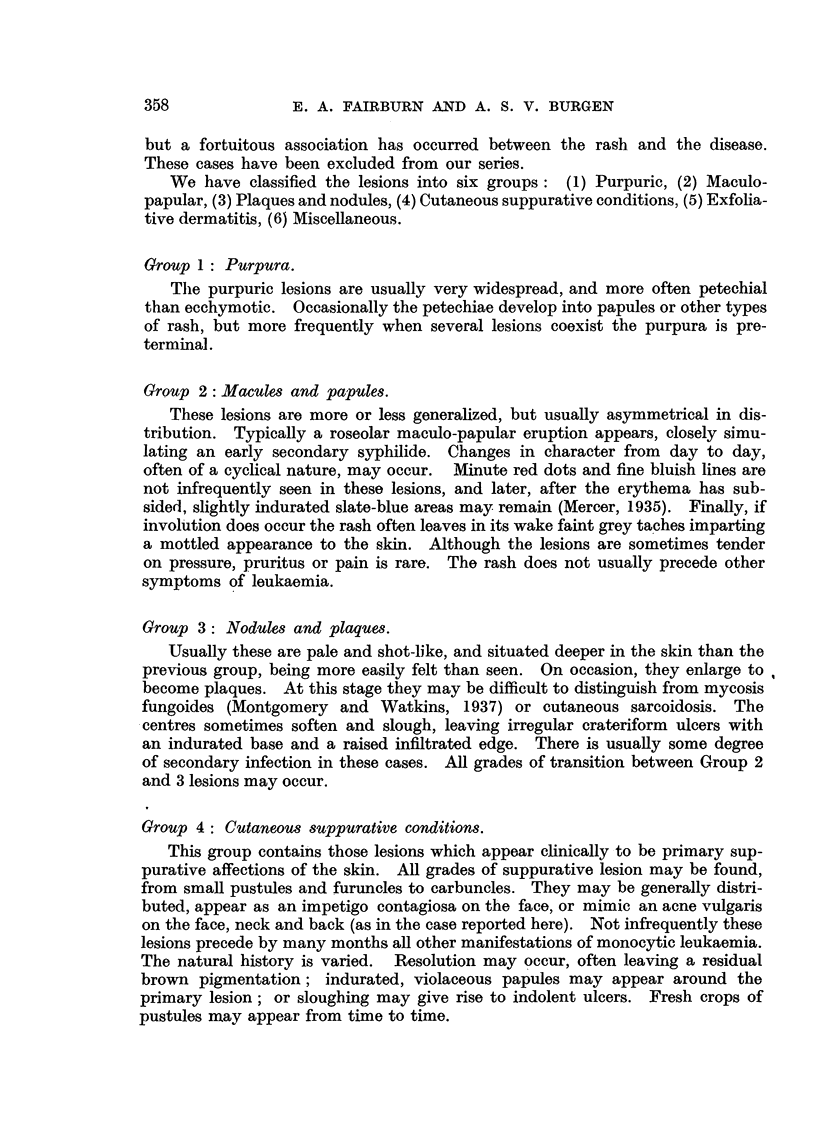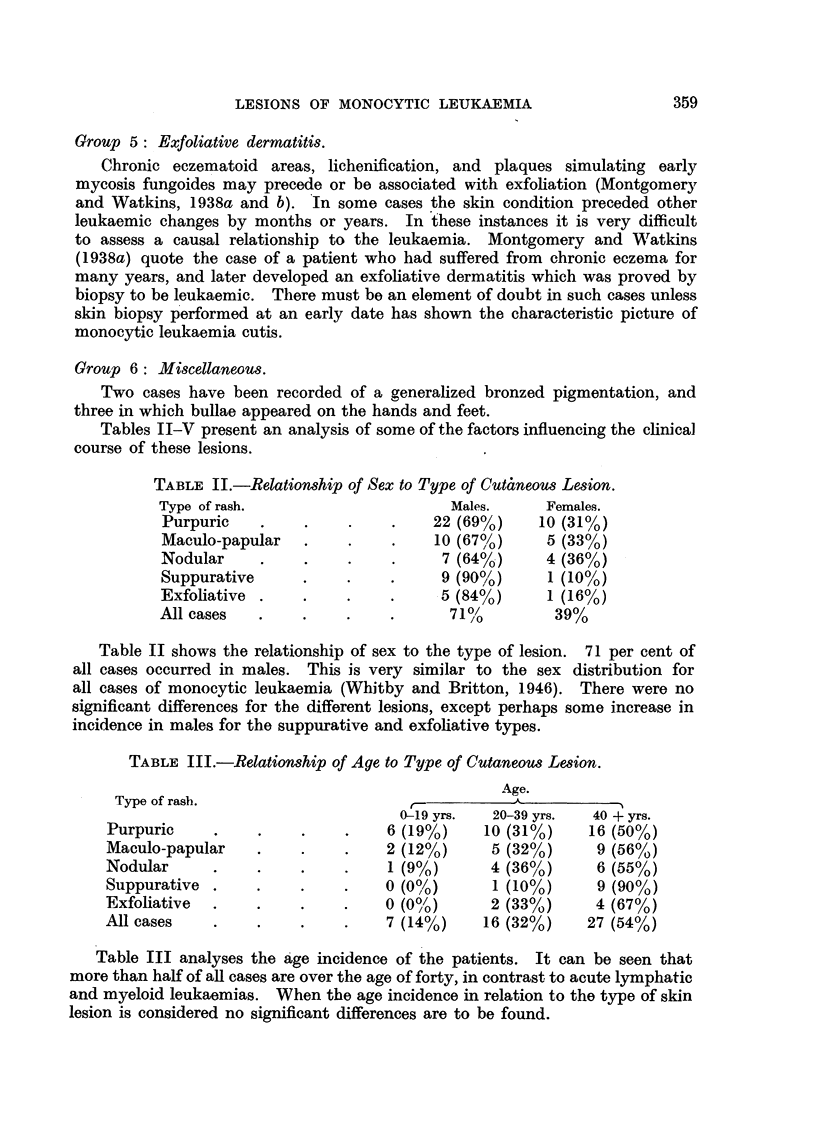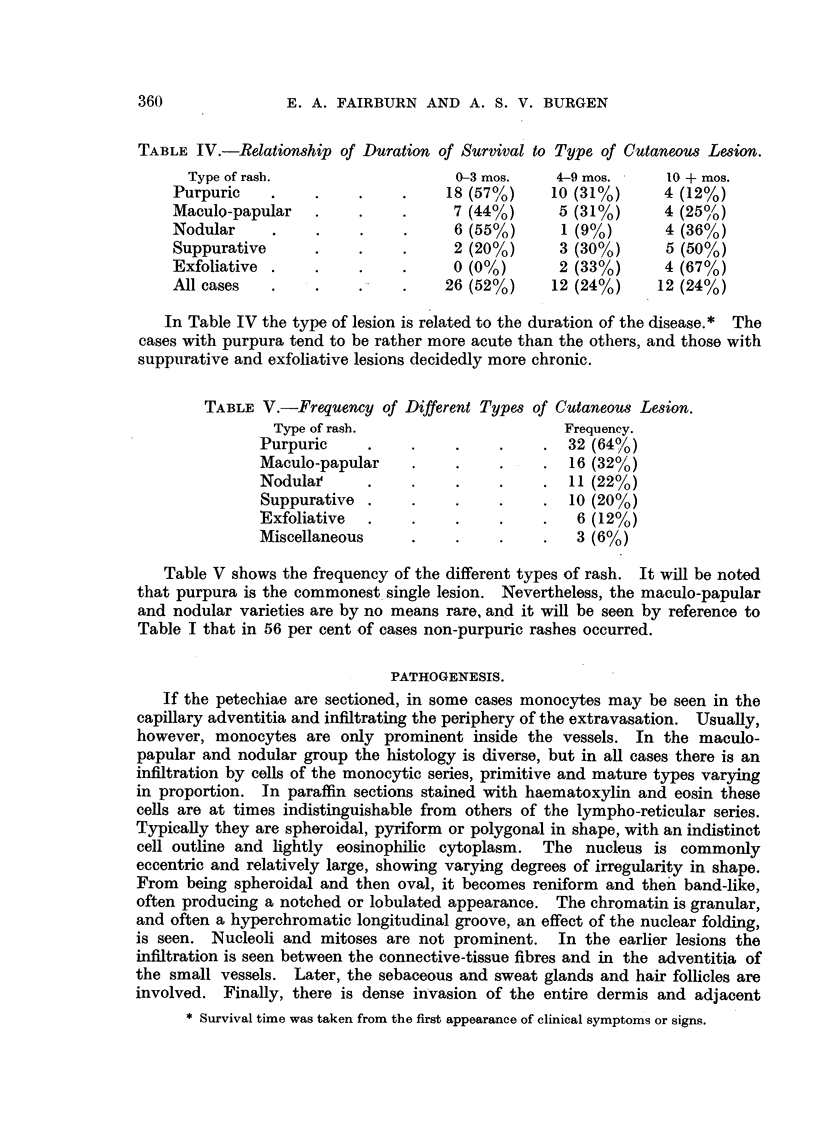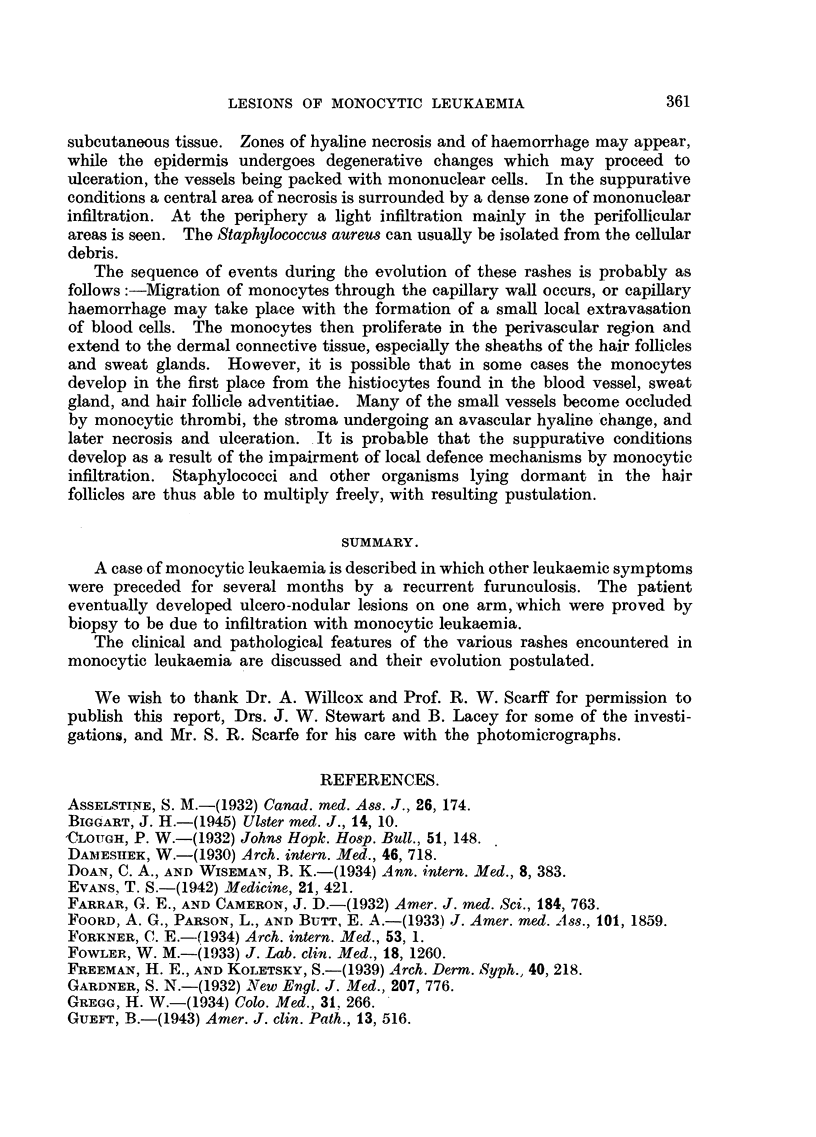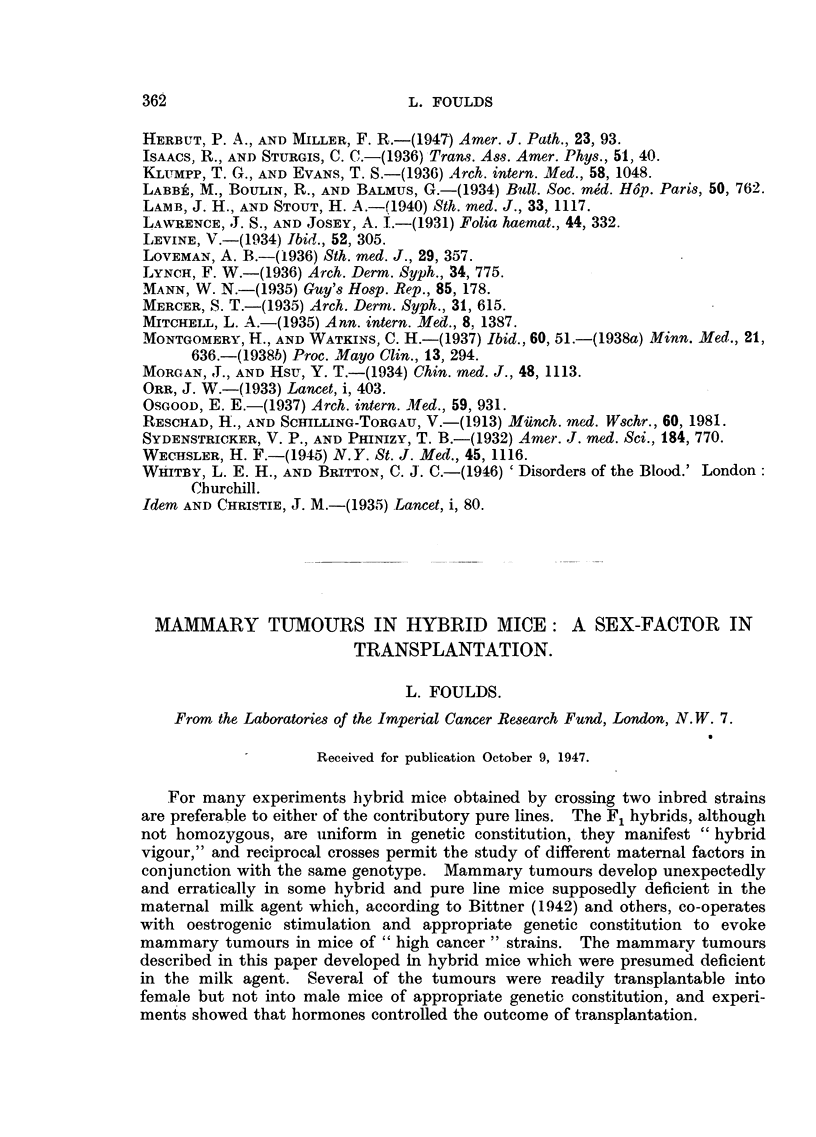# The Skin Lesions of Monocytic Leukaemia

**DOI:** 10.1038/bjc.1947.32

**Published:** 1947-12

**Authors:** E. A. Fairburn, A. S. V. Burgen

## Abstract

**Images:**


### THE SKIN LESIONS OF MONOCYTIC LEUKAEMIA.

E. A. FAIRBURN AND A. S. V. BURGEN.

*From the Wards and the Bland-Sutton Institute of Pathology, Middlesex Hospital.*

Received for publication October 31, 1947.

SINCE 1913, when Reschad and Schilling-Torgau (1913) first distinguished monocytic leukaemia from the lymphatic and myelogenous types, over 250 cases have been reported (Evans, 1942). In their case report Reschad and Schilling-Torgau noted widely distributed nodules in the skin, which, histologically, were packed full of cells similar to those present in the peripheral blood. In most instances subsequent observers have focused their attention on the interesting haematology of the disease, whilst little attention has been paid to the relatively common and often distinctive cutaneous lesions.

In lymphatic and myelogenous leukaemias petechiae in the skin are fairly common. They usually occur in the later stages of the disease, and are indistinguishable from petechiae of other origin. Specific cutaneous changes are rare. In monocytic leukaemia, on the other hand, non-purpuric skin lesions were reported in 36 per cent of patients by Isaacs and Sturgis (1936) and in 10 per cent by Freeman and Koletsky (1939), while of 6 cases of the disease admitted to the Middlesex Hospital in the past 10 years, 2 developed non-purpuric rashes. Further, the purpura of monocytic leukaemia did not always remain petechial. In many reported cases it became raised and infiltrative, and progressed to fully developed nodular or ulcerative lesions. It would appear that the presence of such rashes should rank in diagnostic importance with ulcerative hypertrophic gingivitis. Especially interesting are the rare cases, such as the one reported below, in which a " disseminated furunculosis " or other cutaneous lesion preceded the onset of a leukaemic haematology. The possibility then arises that the leukaemic process has originated in the reticulo-endothelial cells of the skin, and has only later involved locations more intimately concerned with haemopoiesis.

### *Case Report.*

M. F—, male, aged 20. In June, 1945, the patient first suffered an attack of furunculosis involving mainly the shoulders and neck. This gradually subsided during a 6-weeks' stay in hospital. In September, 1945, he joined the Royal Artillery as a gunner, and soon after enlistment had another attack of boils on his back, and to a lesser extent on the neck and face. As before, the lesions gradually healed during a further 8 weeks' hospitalization. In November, 1945, he was drafted to India, where he was stationed at Madras, and later at Bangalore. A few weeks after his arrival in India crops of boils began to appear once more on the shoulders, neck and face. By February, 1946, nodules had developed on the right arm. These never became pustular, but progressed to a brawny thickening, and later formed shallow ulcers. At this time he had a remittent pyrexia and his spleen was observed to be palpable. A swab taken from the ulcers grew numerous colonies of *Staphylococcus aureus*. The leucocyte count was raised to 20,000/cu. mm. with a predominance of neutrophil granulocytes. In consequence a tentative diagnosis of staphylococcal septicaemia was made. However, all attempts to culture an organism from the peripheral blood failed. Moreover, the patient made no response to an adequate course of treatment with local and systemic penicillin.

In successive blood examinations the total leucocyte count rose, and the percentage of mononuclear cells steadily increased. The patient was repeatedly transfused with whole blood, but with no improvement in his condition. In May, 1946, he was returned to England. By this time he was an obvious case of monocytic leukaemia. The ulcers on his arm had become large and irregular, but remained shallow. They were thought to be a type of tropical ulceration. On July 21, 1946, he was transferred to the Middlesex Hospital.

On admission the patient was a pale, wasted youth. The skin of his face, shoulders and neck bore the scars of previous furunculosis. The skin over both tibiae was discoloured by almost confluent petechiae, which were beginning to fade. On the right arm there were a number of brawny, dusky, reddish-brown nodules with a somewhat metallic sheen. These ranged in size from 1–15 cm. in diameter, and the larger of them had undergone a superficial ulceration. The edge of the ulcers was raised, everted, irregular in outline, and paler than the rest of the nodule, the floor being occupied by slough covered by tenacious, purulent exudate (Fig. 1). The ulcers were extremely painful and tender on palpation. Striae atrophicae were present on the flanks and lower back. The gums were moderately hypertrophied and ulcerated, but bled only slightly. The tonsils were very large and pale. A few petechiae were seen on the buccal mucosa. Small, rubbery, mobile lymph glands filled both anterior triangles of the neck, and a few were found in the axillae and groins. The pulse rate was 100/min., and the temperature fluctuated between 99 and 101° F. A soft systolic murmur was heard in the pulmonary area. The spleen was palpable 5 cm. below the costal margin, and the firm liver edge was felt 7 cm. below the right rib margin. The sternum was very tender to percussion. The patient complained of some pain over the left wrist, and on palpation a tender thickening was felt overlying the dorsum of the lower end of the radius.

Blood count (27.vii.46).—Haemoglobin 43 per cent ≡ 6·2 g/100 c.c.; erythrocytes 2·24 millions/cu. mm.; leucocyte count 277,000/cu. mm.

| | Per cent. | Total/cu. mm. |
|---|---|---|
| Differential count: | | |
| Neutrophil polymorphonuclears | 5 | 13,800 |
| Lymphocytes | 1 | 2,700 |
| Monocytes | 90 | 250,000 |
| Metamyelocytes | 2 | 5,500 |
| Myelocytes | 2 | 5,500 |

Blood platelets 24,000/cu. mm.

Film appearances (Fig. 2): The predominant white cell was a typical monocyte, and many showed grooving of the nucleus. There were scanty peroxidase-positive granules in the cytoplasm. Sternal marrow puncture (27.vii.46) (Fig. 3): Nucleated cell count 342,000/cu.mm. (normal 25,000–100,000).

| Differential count : | Per cent. | Normal range %. |
|---|---|---|
| Neutrophil polymorphonuclears | 4 | (20–50) |
| Metamyelocytes | 1·5 | (4–12) |
| Myelocytes | 2·5 | (2–8) |
| Premyelocytes | 1 | (0·5–5) |
| Lymphocytes | 0·5 | (5–20) |
| Monocytes | 88 | (0–5) |
| Monoblasts | 1 | — |
| Late normoblasts | 0·5 | (7–19) |
| Intermediate and early normoblasts | 0·5 | (4–15) |
| Pro-erythroblasts | 0·25 | (0–4) |
| Haemocytoblasts | 0·25 | (0–1) |

A scraping from the edge of an ulcer showed the following cytology (Fig. 4) : Neutrophil polymorphonuclears 4 per cent, lymphocytes 10 per cent, monocytes 81 per cent, monoblasts 4 per cent, plasma cells 1 per cent, and occasional reticulum cells. The monocytes were typical in form and showed scanty peroxidase granules. No leishmaniae, fungi, spirochaetes, actinomycetes, or acid-fast bacilli were seen. A swab from the ulcers showed Gram positive and negative bacilli, and Gram positive cocci. On culture a heavy growth of *Ps. pyocyanea* and a moderate growth of β-haemolytic streptococci and *C. xerosis* was obtained. Wassermann reaction negative. Paul-Bunnell reaction negative to 1/7 (method of Davidsohn).

Radiograms of the left wrist showed early periosteal erosion of the dorsum of the lower end of the radius.

*Progress*.—The ulcers were at first dressed with hypertonic sodium sulphate containing 1 per cent β-phenoxyethanol (Phenoxetol) and later with penicillin cream without any substantial improvement. On July 30, 1946, the patient complained of some abdominal pain, and vomited all he ate, together with a little altered blood. Throughout the next day he vomited bright and altered blood. He became progressively more exhausted and died towards evening.

---

DESCRIPTION OF PLATES.

FIG. 1.—Photograph of the lesions on the arm, showing nodules and large serpiginous ulcers with raised, rolled edge and sloughing base.

FIG. 2.—Photomicrograph of a blood smear, showing typical cells of the monocyte series, some with grooving of the nuclei. Leishman stain. × 1440.

FIG. 3.—Photomicrograph of a sternal marrow smear. Grooved monocytes and promonocytes are seen. Leishman stain. × 1440.

FIG. 4.—Photomicrograph of a scraping from the edge of a skin ulcer, showing grooved monocytes, one of which has a pseudopodium. Leishman stain. × 1440.

FIG. 5.—Photomicrograph of a section of the periphery of an ulcer on the arm, showing normal epithelium with a heavily infiltrated dermis, passing into an area of hyaline necrosis with intense monocytic infiltration and overlying epithelial degneration. Haematoxylin and eosin. × 17.

FIG. 6.—Photomicrograph of a quiescent " furuncle " showing a central zone of hyaline necrosis with heavy infiltration at its edge. In the surrounding region there is a light monocytic infiltration mainly around blood vessels and hair follicles. Haematoxylin and eosin. × 17.

FIG. 7.—Photomicrograph of rectangular area in Fig. 6 showing the infiltration of monocytic cells around the hair follicles and sweat glands. × 125.

1

2

3

4 5

6 7

Fairburn and Burgen.

*Autopsy findings.*

The mouth showed early interdental papillary ulceration. There was marked hypertrophy of the faucial, lingual and naso-pharyngeal tonsils. All the serous membranes showed multiple petechiae and contained varying amounts of blood-stained fluid. There was moderate dilation of the heart, and the myocardium showed early fatty change. The lungs contained multiple recent infarcts up to 4 cm. in diameter. The stomach and intestines were full of altered blood, and the mucosa exhibited many dusky nodular infiltrations up to 1 cm. in diameter. Many of these were superficially ulcerated. The liver weighed 2700 g.; its cut surface was soft and pale, but the pattern was still discernible. The spleen weighed 1350 g., was pale, and had completely lost its pattern. The lymph glands throughout the body showed moderate discrete enlargement. The cut surfaces were greyish-yellow and homogeneous but for areas of recent haemorrhage. The femoral bone-marrow was a uniform greyish-pink hue throughout. Sub-periosteal leukaemic deposits were found in the popliteal region of the right femur and the distal parts of the left ulna and radius.

*Histology.*—The nodular areas showed a heavy infiltration of the entire dermis and superficial subcutaneous tissues with monocytic cells with patchy hyaline eosinophil necrosis and ulceration. The vessels were packed with monocytes (Fig. 5). A section of a subsiding furuncle showed a dense invasion with mono-nuclear cells, while in the less heavily involved edge the hair follicles and sweat glands alone were infiltrated by similar cells. (Figs. 6 and 7). The sternal and femoral bone marrow was extensively replaced by monocytic cells, which were comparatively large and round, oval or pyriform in shape. The cytoplasm was ill-defined. The nucleus was oval, reniform or lobular; it was finely granular with occasional hyperchromatic bars. A distinct nuclear membrane could be seen. There were a moderate number of mitoses but no nucleoli. The lymph glands and spleen had lost their normal architecture, and were diffusely infiltrated with cells of the monocytic series. The liver showed a uniform necrosis of the mid and central zones of the lobule with a heavy peripheral infiltration. The nodules in the intestine were uniformly packed with monocytes.

### DISCUSSION.

A large variety of skin lesions have been described in association with monocytic leukaemia. Most of these are specific in the sense that histological examination of the skin reveals an infiltration, or the local production of cells of the monocytic series. It has been claimed, however, that there is a "toxic" group of lesions (or leukaemides), including haemorrhagic, urticarial, bullous, eczematous and exfoliative types, presumably showing no extravascular monocytic cells. That most of these lesions are really infiltrative has been established by biopsy on repeated occasions (Whitby and Christie, 1935; Montgomery and Watkins, 1937).

We have collected the reports of 50 cases of undoubted Schilling-type monocytic leukaemia in which cutaneous lesions were noticed at some time during the course of the disease (Table I). These are mainly derived from cases published in the English language, but otherwise show no bias. When the skin lesions were of relatively long duration it has sometimes been difficult to believe that any but a fortuitous association has occurred between the rash and the disease. These cases have been excluded from our series.

We have classified the lesions into six groups : (1) Purpuric, (2) Maculo-papular, (3) Plaques and nodules, (4) Cutaneous suppurative conditions, (5) Exfoliative dermatitis, (6) Miscellaneous.

*Group* 1 : *Purpura.*

The purpuric lesions are usually very widespread, and more often petechial than ecchymotic. Occasionally the petechiae develop into papules or other types of rash, but more frequently when several lesions coexist the purpura is preterminal.

*Group* 2 : *Macules and papules.*

These lesions are more or less generalized, but usually asymmetrical in distribution. Typically a roseolar maculo-papular eruption appears, closely simulating an early secondary syphilide. Changes in character from day to day, often of a cyclical nature, may occur. Minute red dots and fine bluish lines are not infrequently seen in these lesions, and later, after the erythema has subsided, slightly indurated slate-blue areas may remain (Mercer, 1935). Finally, if involution does occur the rash often leaves in its wake faint grey taches imparting a mottled appearance to the skin. Although the lesions are sometimes tender on pressure, pruritus or pain is rare. The rash does not usually precede other symptoms of leukaemia.

*Group* 3 : *Nodules and plaques.*

Usually these are pale and shot-like, and situated deeper in the skin than the previous group, being more easily felt than seen. On occasion, they enlarge to become plaques. At this stage they may be difficult to distinguish from mycosis fungoides (Montgomery and Watkins, 1937) or cutaneous sarcoidosis. The centres sometimes soften and slough, leaving irregular crateriform ulcers with an indurated base and a raised infiltrated edge. There is usually some degree of secondary infection in these cases. All grades of transition between Group 2 and 3 lesions may occur.

*Group* 4 : *Cutaneous suppurative conditions.*

This group contains those lesions which appear clinically to be primary suppurative affections of the skin. All grades of suppurative lesion may be found, from small pustules and furuncles to carbuncles. They may be generally distributed, appear as an impetigo contagiosa on the face, or mimic an acne vulgaris on the face, neck and back (as in the case reported here). Not infrequently these lesions precede by many months all other manifestations of monocytic leukaemia. The natural history is varied. Resolution may occur, often leaving a residual brown pigmentation ; indurated, violaceous papules may appear around the primary lesion ; or sloughing may give rise to indolent ulcers. Fresh crops of pustules may appear from time to time.

*Group* 5 : *Exfoliative dermatitis.*

Chronic eczematoid areas, lichenification, and plaques simulating early mycosis fungoides may precede or be associated with exfoliation (Montgomery and Watkins, 1938*a* and *b*). In some cases the skin condition preceded other leukaemic changes by months or years. In these instances it is very difficult to assess a causal relationship to the leukaemia. Montgomery and Watkins (1938*a*) quote the case of a patient who had suffered from chronic eczema for many years, and later developed an exfoliative dermatitis which was proved by biopsy to be leukaemic. There must be an element of doubt in such cases unless skin biopsy performed at an early date has shown the characteristic picture of monocytic leukaemia cutis.

*Group* 6 : *Miscellaneous.*

Two cases have been recorded of a generalized bronzed pigmentation, and three in which bullae appeared on the hands and feet.

Tables II–V present an analysis of some of the factors influencing the clinical course of these lesions.

TABLE II.—*Relationship of Sex to Type of Cutaneous Lesion.*

| Type of rash. | Males. | Females. |
|---|---|---|
| Purpuric . . . . | 22 (69%) | 10 (31%) |
| Maculo-papular . . . | 10 (67%) | 5 (33%) |
| Nodular . . . . | 7 (64%) | 4 (36%) |
| Suppurative . . . | 9 (90%) | 1 (10%) |
| Exfoliative . . . . | 5 (84%) | 1 (16%) |
| All cases . . . . | 71% | 39% |

Table II shows the relationship of sex to the type of lesion. 71 per cent of all cases occurred in males. This is very similar to the sex distribution for all cases of monocytic leukaemia (Whitby and Britton, 1946). There were no significant differences for the different lesions, except perhaps some increase in incidence in males for the suppurative and exfoliative types.

TABLE III.—*Relationship of Age to Type of Cutaneous Lesion.*

| Type of rash. | Age. | | |
|---|---|---|---|
| | 0–19 yrs. | 20–39 yrs. | 40 + yrs. |
| Purpuric . . . . | 6 (19%) | 10 (31%) | 16 (50%) |
| Maculo-papular . . . | 2 (12%) | 5 (32%) | 9 (56%) |
| Nodular . . . . | 1 (9%) | 4 (36%) | 6 (55%) |
| Suppurative . . . . | 0 (0%) | 1 (10%) | 9 (90%) |
| Exfoliative . . . . | 0 (0%) | 2 (33%) | 4 (67%) |
| All cases . . . . | 7 (14%) | 16 (32%) | 27 (54%) |

Table III analyses the age incidence of the patients. It can be seen that more than half of all cases are over the age of forty, in contrast to acute lymphatic and myeloid leukaemias. When the age incidence in relation to the type of skin lesion is considered no significant differences are to be found.

TABLE IV.—*Relationship of Duration of Survival to Type of Cutaneous Lesion.*

| Type of rash. | 0–3 mos. | 4–9 mos. | 10 + mos. |
|---|---|---|---|
| Purpuric | 18 (57%) | 10 (31%) | 4 (12%) |
| Maculo-papular | 7 (44%) | 5 (31%) | 4 (25%) |
| Nodular | 6 (55%) | 1 (9%) | 4 (36%) |
| Suppurative | 2 (20%) | 3 (30%) | 5 (50%) |
| Exfoliative | 0 (0%) | 2 (33%) | 4 (67%) |
| All cases | 26 (52%) | 12 (24%) | 12 (24%) |

In Table IV the type of lesion is related to the duration of the disease.* The cases with purpura tend to be rather more acute than the others, and those with suppurative and exfoliative lesions decidedly more chronic.

TABLE V.—*Frequency of Different Types of Cutaneous Lesion.*

| Type of rash. | Frequency. |
|---|---|
| Purpuric | 32 (64%) |
| Maculo-papular | 16 (32%) |
| Nodular | 11 (22%) |
| Suppurative | 10 (20%) |
| Exfoliative | 6 (12%) |
| Miscellaneous | 3 (6%) |

Table V shows the frequency of the different types of rash. It will be noted that purpura is the commonest single lesion. Nevertheless, the maculo-papular and nodular varieties are by no means rare, and it will be seen by reference to Table I that in 56 per cent of cases non-purpuric rashes occurred.

### PATHOGENESIS.

If the petechiae are sectioned, in some cases monocytes may be seen in the capillary adventitia and infiltrating the periphery of the extravasation. Usually, however, monocytes are only prominent inside the vessels. In the maculo-papular and nodular group the histology is diverse, but in all cases there is an infiltration by cells of the monocytic series, primitive and mature types varying in proportion. In paraffin sections stained with haematoxylin and eosin these cells are at times indistinguishable from others of the lympho-reticular series. Typically they are spheroidal, pyriform or polygonal in shape, with an indistinct cell outline and lightly eosinophilic cytoplasm. The nucleus is commonly eccentric and relatively large, showing varying degrees of irregularity in shape. From being spheroidal and then oval, it becomes reniform and then band-like, often producing a notched or lobulated appearance. The chromatin is granular, and often a hyperchromatic longitudinal groove, an effect of the nuclear folding, is seen. Nucleoli and mitoses are not prominent. In the earlier lesions the infiltration is seen between the connective-tissue fibres and in the adventitia of the small vessels. Later, the sebaceous and sweat glands and hair follicles are involved. Finally, there is dense invasion of the entire dermis and adjacent

* Survival time was taken from the first appearance of clinical symptoms or signs.

subcutaneous tissue. Zones of hyaline necrosis and of haemorrhage may appear, while the epidermis undergoes degenerative changes which may proceed to ulceration, the vessels being packed with mononuclear cells. In the suppurative conditions a central area of necrosis is surrounded by a dense zone of mononuclear infiltration. At the periphery a light infiltration mainly in the perifollicular areas is seen. The *Staphylococcus aureus* can usually be isolated from the cellular debris.

The sequence of events during the evolution of these rashes is probably as follows :—Migration of monocytes through the capillary wall occurs, or capillary haemorrhage may take place with the formation of a small local extravasation of blood cells. The monocytes then proliferate in the perivascular region and extend to the dermal connective tissue, especially the sheaths of the hair follicles and sweat glands. However, it is possible that in some cases the monocytes develop in the first place from the histiocytes found in the blood vessel, sweat gland, and hair follicle adventitiae. Many of the small vessels become occluded by monocytic thrombi, the stroma undergoing an avascular hyaline change, and later necrosis and ulceration. It is probable that the suppurative conditions develop as a result of the impairment of local defence mechanisms by monocytic infiltration. Staphylococci and other organisms lying dormant in the hair follicles are thus able to multiply freely, with resulting pustulation.

### SUMMARY.

A case of monocytic leukaemia is described in which other leukaemic symptoms were preceded for several months by a recurrent furunculosis. The patient eventually developed ulcero-nodular lesions on one arm, which were proved by biopsy to be due to infiltration with monocytic leukaemia.

The clinical and pathological features of the various rashes encountered in monocytic leukaemia are discussed and their evolution postulated.

We wish to thank Dr. A. Willcox and Prof. R. W. Scarff for permission to publish this report, Drs. J. W. Stewart and B. Lacey for some of the investigations, and Mr. S. R. Scarfe for his care with the photomicrographs.

### REFERENCES.

Asselstine, S. M.—(1932) *Canad. med. Ass. J.*, **26**, 174.
Biggart, J. H.—(1945) *Ulster med. J.*, **14**, 10.
Clough, P. W.—(1932) *Johns Hopk. Hosp. Bull.*, **51**, 148.
Dameshek, W.—(1930) *Arch. intern. Med.*, **46**, 718.
Doan, C. A., and Wiseman, B. K.—(1934) *Ann. intern. Med.*, **8**, 383.
Evans, T. S.—(1942) *Medicine*, **21**, 421.
Farrar, G. E., and Cameron, J. D.—(1932) *Amer. J. med. Sci.*, **184**, 763.
Foord, A. G., Parson, L., and Butt, E. A.—(1933) *J. Amer. med. Ass.*, **101**, 1859.
Forkner, C. E.—(1934) *Arch. intern. Med.*, **53**, 1.
Fowler, W. M.—(1933) *J. Lab. clin. Med.*, **18**, 1260.
Freeman, H. E., and Koletsky, S.—(1939) *Arch. Derm. Syph.*, **40**, 218.
Gardner, S. N.—(1932) *New Engl. J. Med.*, **207**, 776.
Gregg, H. W.—(1934) *Colo. Med.*, **31**, 266.
Gueft, B.—(1943) *Amer. J. clin. Path.*, **13**, 516.

HERBUT, P. A., AND MILLER, F. R.—(1947) *Amer. J. Path.*, **23**, 93.
ISAACS, R., AND STURGIS, C. C.—(1936) *Trans. Ass. Amer. Phys.*, **51**, 40.
KLUMPP, T. G., AND EVANS, T. S.—(1936) *Arch. intern. Med.*, **58**, 1048.
LABBÉ, M., BOULIN, R., AND BALMUS, G.—(1934) *Bull. Soc. méd. Hôp. Paris*, **50**, 762.
LAMB, J. H., AND STOUT, H. A.—(1940) *Sth. med. J.*, **33**, 1117.
LAWRENCE, J. S., AND JOSEY, A. I.—(1931) *Folia haemat.*, **44**, 332.
LEVINE, V.—(1934) *Ibid.*, **52**, 305.
LOVEMAN, A. B.—(1936) *Sth. med. J.*, **29**, 357.
LYNCH, F. W.—(1936) *Arch. Derm. Syph.*, **34**, 775.
MANN, W. N.—(1935) *Guy's Hosp. Rep.*, **85**, 178.
MERCER, S. T.—(1935) *Arch. Derm. Syph.*, **31**, 615.
MITCHELL, L. A.—(1935) *Ann. intern. Med.*, **8**, 1387.
MONTGOMERY, H., AND WATKINS, C. H.—(1937) *Ibid.*, **60**, 51.—(1938*a*) *Minn. Med.*, **21**, 636.—(1938*b*) *Proc. Mayo Clin.*, **13**, 294.
MORGAN, J., AND HSU, Y. T.—(1934) *Chin. med. J.*, **48**, 1113.
ORR, J. W.—(1933) *Lancet*, i, 403.
OSGOOD, E. E.—(1937) *Arch. intern. Med.*, **59**, 931.
RESCHAD, H., AND SCHILLING-TORGAU, V.—(1913) *Münch. med. Wschr.*, **60**, 1981.
SYDENSTRICKER, V. P., AND PHINIZY, T. B.—(1932) *Amer. J. med. Sci.*, **184**, 770.
WECHSLER, H. F.—(1945) *N.Y. St. J. Med.*, **45**, 1116.
WHITBY, L. E. H., AND BRITTON, C. J. C.—(1946) 'Disorders of the Blood.' London : Churchill.
*Idem* AND CHRISTIE, J. M.—(1935) *Lancet*, i, 80.